# Meta-analysis of Chicken – Salmonella infection experiments

**DOI:** 10.1186/1471-2164-13-146

**Published:** 2012-04-24

**Authors:** Marinus FW te Pas, Ina Hulsegge, Dirkjan Schokker, Mari A Smits, Mark Fife, Rima Zoorob, Marie-Laure Endale, Johanna MJ Rebel

**Affiliations:** 1 Animal Breeding and Genetics Centre (ABGC), Wageningen UR Livestock Research, Animal Sciences Group, Wageningen University and Research Centre, P.O. Box 65, 8200 AB, Lelystad, The Netherlands; 2 Central Veterinary Institute - Department of Infectious Biology, Animal Sciences Group, Wageningen University and Research Centre, P.O. Box 65, 8200 AB, Lelystad, The Netherlands; 3 Institute for Animal Health, Genetics & Genomics group, Compton, Berkshire, UK; 4 INSERM UMR-S 945, Institut Fédératif de Recherches (IFR) 113, department of Immunité-Cancer-Infection, Hôpital Pitié-Salpêtrière, 83 Bld de l'Hôpital, Bâtiment CERVI, 75651, Paris, Cédex 13, France; 5 INRA, AgroParisTech, UMR1313 Animal Genetics and Integrative Biology, F-78350, Jouy-en-Josas, France

## Abstract

**Background:**

Chicken meat and eggs can be a source of human zoonotic pathogens, especially Salmonella species. These food items contain a potential hazard for humans. Chickens lines differ in susceptibility for Salmonella and can harbor Salmonella pathogens without showing clinical signs of illness. Many investigations including genomic studies have examined the mechanisms how chickens react to infection. Apart from the innate immune response, many physiological mechanisms and pathways are reported to be involved in the chicken host response to Salmonella infection. The objective of this study was to perform a meta-analysis of diverse experiments to identify general and host specific mechanisms to the Salmonella challenge.

**Results:**

Diverse chicken lines differing in susceptibility to Salmonella infection were challenged with different Salmonella serovars at several time points. Various tissues were sampled at different time points post-infection, and resulting host transcriptional differences investigated using different microarray platforms. The meta-analysis was performed with the R-package metaMA to create lists of differentially regulated genes. These gene lists showed many similarities for different chicken breeds and tissues, and also for different Salmonella serovars measured at different times post infection. Functional biological analysis of these differentially expressed gene lists revealed several common mechanisms for the chicken host response to Salmonella infection. The meta-analysis-specific genes (i.e. genes found differentially expressed only in the meta-analysis) confirmed and expanded the biological functional mechanisms.

**Conclusions:**

The meta-analysis combination of heterogeneous expression profiling data provided useful insights into the common metabolic pathways and functions of different chicken lines infected with different Salmonella serovars.

## Background

Chicken meat and eggs for human consumption can be contaminated with several Salmonella species, and therefore chicken-derived food products can be regarded as a source of human zoonotic pathogens. Although proper food preparation should kill the pathogens, the food items contain a potential hazard for humans. In chicken both acute fatal and chronic Salmonellosis occurs depending upon the infecting Salmonella serovar [1-4]. Broad host range Salmonella serovars used most often in studies – including the studies used for this meta-analysis, S. Typhimurium and *S.* Enteritidis, do not cause fatal infections when chickens older than one day post hatch are orally challenged. Chickens can harbor Salmonella pathogen without showing clinical signs of illness [3,5]. Many investigations have examined the mechanisms how chickens react to infection, the mechanism of transfer to humans and host immunity to infection [3,6].

Diverse host species may react differently to Salmonella infection [7]. While one-day old chickens may succumb to broad host range Salmonella infection, older chickens often show no clinical signs. Furthermore, specific chicken lines have been shown to differ in their susceptibility for Salmonella [8-11]. These clear genetic differences in susceptibility may be due to pleiotropic effects, or to unknown selection-related mechanisms. In the last decade, gene expression profiling studies using microarrays have been widespread in animal genomics and have enabled researchers to monitor the effects of pathogens on host cells and tissues with the aim of gaining insights into the molecular mechanisms that are involved in the host-pathogen interactions. Several genes involved in Salmonella susceptibility in chicken have been determined [12-17]. Apart from the innate immune response, many physiological mechanisms and pathways were reported to be involved in the chicken host response to Salmonella infection which are also active in uninfected cells, including energy metabolism, cell shape, and others [18-20].

Each of these independent experiments showed how individual hosts within the specific experimental conditions reacted to Salmonella infection. Meta-analysis of these experiment may reveal a common genetic background for the chicken host reaction to the Salmonella infection. Furthermore, the age-related differences in the mechanisms and the outcome of the host immune-response to Salmonella infection suggests that different immune-reactions are possible, and are likely to be age related [21,22]. Taken together this indicates a complex interplay between chicken host genetics and Salmonella serovars [3,10,11,18,23].

Meta-analysis methods integrate results of independent studies creating very large datasets with increased statistical power [24,25]. It allows a more objective appraisal of evidence than individual studies, and has been widely used to interpret contradictory results from diverse studies. Furthermore, this analysis method overcomes the problem of reduced statistical power associated with studies of small sample size (reviewed by [26,27]. Such methods enable analyses at a higher level than possible on the individual datasets. Host-specific general mechanisms can be determined in addition to mechanisms operating under specific conditions. Thus, using previously published individual datasets we were able to highlight new results that contribute to understanding of common disease mechanisms and physiology. Different experiments were performed under the umbrella of a large EU-funded project called SABRE - Cutting Edge Genomics for Sustainable Animal Breeding [9,21,22,28,29]. This meta-analysis brings the individual studies together offering the potential to highlight new host-pathogen interaction mechanisms and elucidate possible general host-response mechanisms. The objective of this study was to determine the general chicken host response to Salmonella infection independent of age of the chicken, age at infection and, time post infection and independent of host response time post-infection. The results indicate several common chicken host reaction mechanisms to Salmonella infection.

## Methods

### Animals and Salmonella challenges

#### Experiment 1

The original animal experiment was described by Fife et al. [29]. In short, two inbred chicken lines differing in susceptibility to gut pathogens (lines N and 6, with line 6 more resistant than line N, [29]) were at three weeks of age orally infected with 5.1x10^7^-1.97x10^8^ cfu *S.* Typhimurium according to the method of Barrow et al. [30]. The caecal tonsils and spleens were sampled at 2, 3, and 4 days post infection, (n = 10), and four birds at each time point were used as uninfected controls. Total RNA for these samples was isolated and used for hybridization to the 20.6 K chicken oligo array (ARK genomics; http://www.ark-genomics.org/) microarrays. Infection and infection clearance was determined by ceacal counts of *S.* Typhimurium *(cfu 10*^*6*^ *l)* and differences between the lines investigated. A total of 32 microarrays per line were obtained.

#### Experiment 2

The original experiment was described by Schokker et al. [31] (GEO data: GSE27069). In short, three commercial chicken lines differing for Salmonella sensitivity were orally infected with 10^5^ cfu *S.* Enteritidis at the day of hatch. The jejunum was sampled at 8 h and days 1 and 2 post infection, 10 animals each, of which 5 were used for microarray analysis. A reference pool was created from 0.33, 1 and 2 days post infection birds, for all three lines together, as well as control and infected birds. Infection was checked by body weight and liver weight gain and liver clearance, and cloaca swaps. Total RNA was isolated and hybridized to the same microarrays as experiment 1. A total of 45 microarrays were obtained [31].

#### Experiment 3

The original experiment was described by Schokker et al. [16,28] (ArrayExpress data: E-MEXP-042). In short, chickens were challenged orally at the day of hatch with 10^5^ cfu *S.* Enteritidis. The jejunum was sampled at 8 h, and 1, 2, 4, 8, 12, and 21 days post infection, 5 animals each for both control and infected situation. Infection was checked by body weight and liver weight gain and liver clearance. Total RNA was isolated and single color hybridized against Agilent chicken microarrays. A total of 70 microarrays were obtained [16,21].

#### Experiment 4

The original experiment was described by van Hemert et al. [21] (GEO data: GSE3702). In short, two chicken lines differing in growth rate and Salmonella sensitivity were orally infected with 10^5^ cfu of *S.* Enteritidis at one day of age and jejunum samples were taken after 24 h. A non-infected control was used, 5 chicken each. Total RNA was isolated and hybridized against Affymetrix chicken microarrays using group comparison. A total of four microarrays were obtained [21].

### Meta-analysis methodology

#### Pre-processing microarray data

The microarray data pre-processing was carried out using functions from the LIMMA package (version 3.2.1) [32]. The quality of the arrays was evaluated through several diagnostic plots. The “normexp” method [33] was used for background correction, followed by normalization within individual microarrays using the default “print tip loess” method and normalization between arrays using the “quantile” method. The background correction was set to: offset = 50. The offset can be used to add a constant to the intensities before log-transforming, so that the log-ratios are shrunk towards zero at the lower intensities. This may eliminate or reverse the usual 'fanning' of log-ratios at low intensities associated with local background subtraction. Areas with higher than average background were removed from the results. Bad hybridization always removed whole microarrays. Especially in experiment 1 this removed parts of the results. After normalization 20 slides of experiment 1 and one slide of Experiment 2 were deleted due to poor quality hybridization. This will inevitably affect the results, but this procedure ensures that only good quality data were used.

#### Meta-analysis

**Figure 1:**
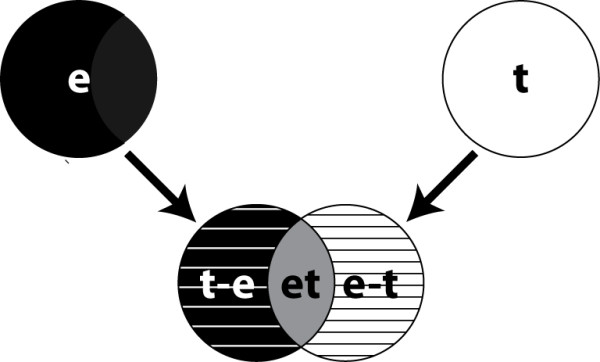
**Overview experiment and time analyses.** For both IDD and DE experiment (e) and time (t) gene lists were generated. Thereafter different subsets were extracted, unique time genes (t-e), unique experiment genes (e-t), and overlap between (e) and (t) (et).

Meta-analysis was carried out using the directpvalcombi function from the metaMA package (Meta-analysis for MicroArrays) (version 1.1) in R [24]. The input for the meta-analysis were the individual microarrays of all experiments. The meta-analysis produced lists of gene names with differential expression under specific conditions. The lists of genes were grouped in (1) **DE**: the list of Differentially Expressed genes (i.e. in the experiments and in the meta-analysis), and (2) **IDD** (Integration Driven Discoveries): the list of genes that were determined differentially expressed in the meta-analysis that were not identified in any of the individual studies alone (i.e. new differentially expressed genes). For both DE and IDD gene lists (e) experiment (i.e. 4 studies) and (t) time (14 studies) were generated. Subsequently from these (e) and (t) the following groups were also extracted, namely (et): overlap between the (e) and (t) groups, (e-t): genes unique in (e), and (t-e): genes unique in (t) (Figure 1). The groups included results from the different tissues. Since the analyses focus on expression differences related to Salmonella infection no interaction with tissue-specific gene expression can be expected.

### Functional bioinformatics analyses

The lists of differentially expressed genes were analyzed for biological functionalities using the DAVID (The Database for Annotation, Visualization and Integrated Discovery) software [34-36], version 6.6. The gene lists were analyzed against the gene list of the human genome since the annotation of the human genome, especially with physiological data, is more advanced than the chicken genome. Therefore, all genes were converted to human Entrez identifiers. The false discovery rate, multiple testing correction for statistical significance [37], and the fold enrichment analyses were manually included for all analyses. The tissue-specific profiles and functional annotations and clusterings of the gene lists were investigated.

## Results

### Meta-analysis

**Figure 2:**
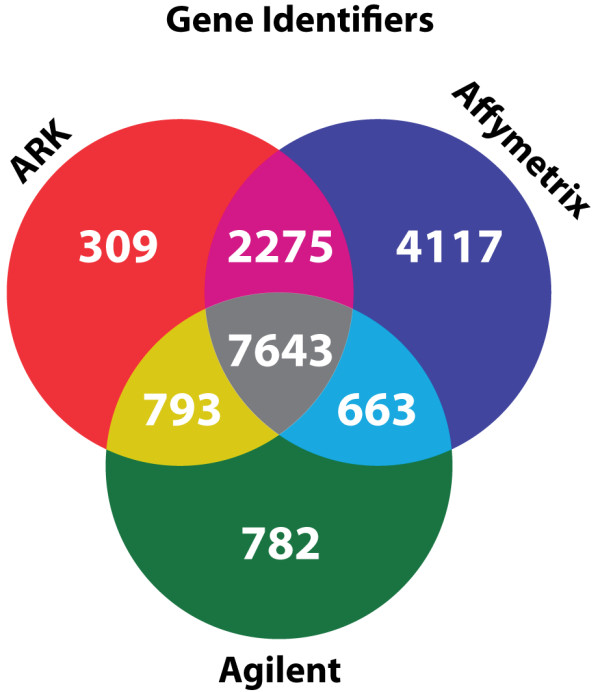
**Visualization of the overlap between the microarray platforms.** For the three different platforms, ARK-genomics, Affymetrix and Agilent, all probes were mapped to human entrez gene identifiers (EGIDs). Subsequently the overlap between all platforms was calculated.

Due to the platform differences used in the individual studies the number of genes available in all studies was reduced as expressed in Figure 2. The Figure shows that 7,643 genes were common to all microarray platforms and thus available for meta-analysis over all studies. Different platforms may use different probes for the same genes, and the probes may differ in hybridization characteristics. However, since differential expression of genes was measured within a platform for each of the experiments this will not affect the meta-analysis.

**Figure 3:**
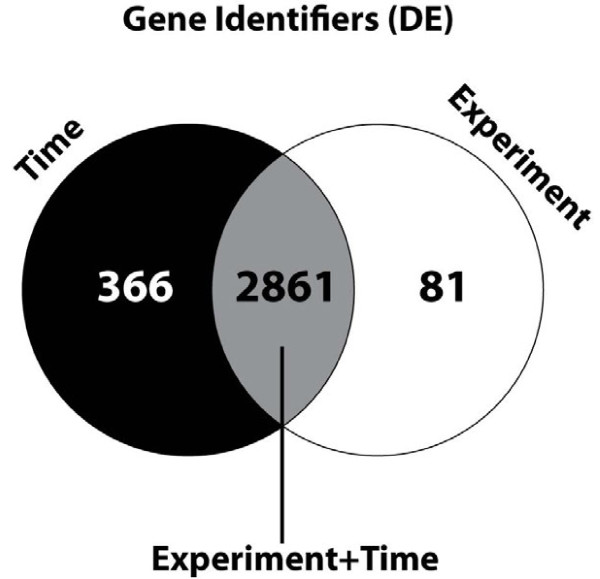
**Visualization of the differentially expressed genes of the DE-group.** The (e), (t), and (e + t) categories show large overlap between the categories. The lists of genes were grouped in (1) DE: the Number of Differentially Expressed genes (i.e. in the experiments and in the meta-analysis), and (2) IDD (Integration Driven Discoveries): the number of genes that were determined differentially expressed in the meta-analysis that were not identified in any of the individual studies alone (i.e. new differentially expressed genes). These two groups were studied in detail in five categories each: (e): experiment - i.e. 4 studies, (t): time after infection – 14 different time point post-infection, (et): overlap between the (e) and (t) groups, (e-t): genes unique in (e), and (t-e): genes unique in (t).

The results of the meta-analysis DE-group were expressed in several lists of genes with regulated expression in more than one or all experiments (Table 1 – the Table with the gene lists is Additional file 1). The Table shows that approximately 3,000 genes are differently expressed, irrespective of age of infection or Salmonella serovar of infection. Differential expression was between control and infected animals of the same age. Furthermore, most differently expressed genes were the same in all experiments irrespective of sampling time point post-infection (Figure 3). The Figure shows that the large majority of the genes are shared by the (e) and the (t) categories.

**Table 1:** Number of differentially expressed genes per group

**Category**	**DE**	**IDD**
**e**	2942	85
**t**	3227	378
**et**	2861	61
**e-t**	81	24
**t-e**	366	317

Number of differentially expressed genes in the groups Differentially Expressed (DE) genes and Integration Driven Discoveries (IDD), the latter of which is specific for genes only found in the meta-analysis. Categories depend on the experimental differences: (e) experiment, (t) time post infection, (et) both (e) and (t), (e-t) experiment specific, and (t-e) time specific.

**Figure 4:**
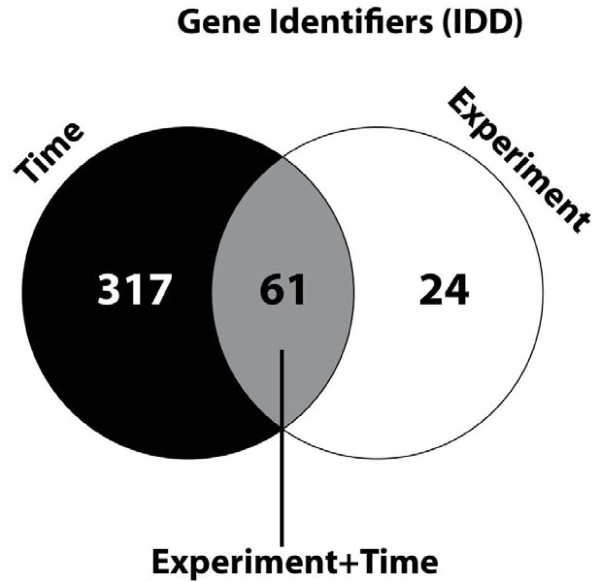
**Visualization of the differentially expressed genes of the IDD-group.** The (e), (t), and (e + t) categories showing overlap between the categories. The lists of genes were grouped in (1) DE: the Number of Differentially Expressed genes (i.e. in the experiments and in the meta-analysis), and (2) IDD (Integration Driven Discoveries): the number of genes that were determined differentially expressed in the meta-analysis that were not identified in any of the individual studies alone (i.e. new differentially expressed genes). These two groups were studied in detail in five categories each: (e): experiment - i.e. 4 studies, (t): time after infection – 14 different time point post-infection, (et): overlap between the (e) and (t) groups, (e-t): genes unique in (e), and (t-e): genes unique in (t).

The IDD group (i.e. the list of genes found only differently expressed in the meta-analysis) genes were predominantly in the (t) category (Figure 4). A few genes were differently expressed in the meta-analysis only for the (e) category.

### Functional bioinformatics analyses

The gene lists were analyzed for biological functional groups using the DAVID software. First the expression profile of differentially expressed genes of the DE-group was compared with normal tissue-specific expression profiles from the same time points and the same tissue type (Table 2). Significant results were only obtained for the DE group, (e), (t), and (et) categories. The Table indicates that the sampled intestinal tissue showed expression profiles related to a number of different tissue types. The most significant tissue expression profile is epithelium. This cell type is abundantly present in intestinal tissue. Several other cell types and tissues also showed similarities for tissue-specific expression profiles, some of them not relevant in intestine (data not shown).

**Table 2:** Tissue specificity of differentially expressed gene profiles

	**Experiment (e)**				**(et)**				**Time (t)**			
**Tissue**	**N**	**FE**	**Benjamini**	**FDR**	**N**	**FE**	**Benjamini**	**FDR**	**N**	**FE**	**Benjamini**	**FDR**
Epithelium	636	1.56	1.50E-34	4.99E-34	616	1.56	1.36E-32	4.54E-32	682	1.53	1.20E-34	3.92E-34
Liver	476	1.48	2.68E-19	1.79E-18	464	1.48	7.21E-19	4.82E-18	516	1.46	2.04E-20	1.33E-19
Brain	1443	1.17	4.35E-16	4.36E-15	1400	1.17	1.55E-14	1.55E-13	1566	1.16	5.17E-16	5.07E-15
Skin	360	1.28	6.81E-06	1.59E-04	351	1.28	8.22E-06	1.92E-04	392	1.28	2.63E-06	6.02E-05
Lymph	152	1.46	3.10E-05	8.28E-04	148	1.47	4.44E-05	0.001	157	1.38	3.98E-04	0.016
Bone marrow	160	1.41	1.76E-04	0.005	158	1.43	8.78E-05	0.003	178	1.43	1.45E-05	0.000
Muscle	170	1.34	0.001	0.049	167	1.35	9.43E-04	0.035	189	1.36	1.46E-04	0.005
Cajal-Retzius cell	53	1.75	0.001	0.056	52	1.77	0.001	0.055	56	1.69	0.002	0.079
Skeletal muscle	123	1.38	0.003	0.170	122	1.41	0.002	0.079	141	1.45	0.000	0.004
Colon	229	1.25	0.003	0.179	224	1.26	0.003	0.157	238	1.19	0.035	2.520
Fetal brain cortex	55	1.65	0.004	0.208	54	1.67	0.004	0.196	59	1.62	0.004	0.181
Heart	119	1.34	0.013	0.791	112	1.29	0.043	3.784	130	1.34	0.008	0.433
Renal cell carcinoma	21	2.17	0.017	1.111	21	2.23	0.013	0.777	21	1.99	0.042	3.417
Lung	453	1.13	0.030	2.239	447	1.15	0.013	0.811	504	1.15	0.005	0.249
Hepatoma	54	1.49	0.038	2.953	54	1.54	0.023	1.605	59	1.49	0.026	1.717
Embryonal rhabdomyosarcoma	9	3.34	0.043	3.425	9	3.43	0.036	2.866				
Kidney					265	1.18	0.034	2.526	300	1.19	0.013	0.767
Fetal liver					48	1.53	0.042	3.529				
Teratocarcinoma									120	1.29	0.034	2.585
Aorta									36	1.66	0.035	2.768

To investigate cell types and tissues related to the differentially expressed gene profiles the DAVID software compared the lists of differentially expressed genes with normal physiologic expression of tissue-specific gene profiles. N is the number of differently expressed genes found in a tissue, FE is Fold Enrichment, Benjamini is the P-value after correction for multiple testing, and FDR is False Discovery rate.

**Table 3:** Clustering of gene lists using functional annotations

**Enrichment score (e)**	**Enrichment score (et)**	**(e)**^**1**^	**(et)**^**1**^	**(t-deduced)**^**1**^	**Content Focus**
10.09	10.46	1	1	1	Lumen of organelles, specifically the nucleus
8.65	8.06	2	3	3	ATP / nucleotide binding; phosphorylation, (ser, thr) kinase, transferase, S_TKc
8.15	8.14	3	2	2	Mitochondrion (outer and inner membranes)
6.11	5.47	4	7	4-7	SH3 protein domain
5.94	6.30	5	5	5	Mitochondrion
5.31	5.93	6	6	6	Macromolecules, specifically protein catabolism, including UBL mechanism
5.00	6.42	7	4, 24	4-7, ± 20	Macromolecules / protein transport, especially import in nucleus / localization
4.83	4.28	8	8	8	Non-membrane bound organelles and cytoskeleton
4.31	3.95	9	10	9-10	Cell cycle (process)
4.12	3.57	10	13	±15	actin cytoskeleton (binding)
3.94	3.18	11	19	?	Protein folding / Chaperone protein
3.90	3.85	12	11	11	Angiogenesis
3.73	3.69	13	12	12	Ubl conjugation
XXX	3.56	X	14	<10	Tyrosine phosphorylation
3.68	3.44	14	15	15	Endoplasmic reticulum
3.63	XXX	15	X	X	Ubiquitin / proteasome proteolysis
3.56	3.95	16	9	4-7	Transcription
3.52	3.22	17	17	17	GTPase activity
3.03	3.22	18	18	18	Muscle morphology
3.01	XXX	19	X	X	Intracellular vesicles
2.85	2.14	21	36	?	Cell-cell contacts
2.82	2.98	22	20	20	Protein modification and metabolism, including proteolysis
2.75	XXX	23	X	X	Protein domain WD (repeat)
2.70	2.31	24	31	?	
2.70	2.68	25	23	23	Apoptosis
2.69	2.72	26	22	±20	RRM (RNA recognition motif)
2.64	2.27	27	32	?	Macromolecule complexes, especially protein complexes
2.45	2.07	31	38	±40	Lysosome
2.40	XXX	34	X	X	Cell movement
2.37	2.21	35	33	33	Mitochondrion / organelle outer membrane
2.21	1.67	36	52	?	Nucleotide binding via P-loop domain
2.08	1.94	38	41	41	Nuclear pore / RNA transport
2.05	2.14	39	37	37	Negative regulation of biosynthesis (nucleic acid, protein, macromolecules)
2.05	2.04	40	39	39	Cellular response to diverse stimuli
1.03	2.2 - 1.55	126	35 + 61	<40	Muscle proteins, skeletal muscle morphology proteins

Clustering of the functional annotations of the gene lists of the differential expression (DE) group, i.e. the Number of Differentially Expressed genes (i.e. in the experiments and in the meta-analysis), and categories: (e): experiment - i.e. 4 studies, (t): time after infection – 14 different time point post-infection, and (et): overlap between the (e) and (t) groups. The t-values were deduced from the other groups because the number of data was too large for the DAVID software to analyze directly. 1: Number of the cluster within group.

The results of the differently expressed gene lists were then analyzed for functional biological mechanisms. The results are shown in Additional file 2. The results showed lists of biological functions for the (e) and the (t) categories of the DE group. Moreover, the (et) category showed that both lists were largely similar, and the top of the lists were even identical. The top of the lists indicated that phosphorylation of proteins, acetylation in the cytoplasm and lumen of other cellular components, and ATP consuming processes were important biological mechanisms during chicken host reaction to Salmonella infection. The meta-analysis showed additional significant results for both the experiment (e-t) and the time (t-e) categories of the phosphoprotein biological function during chicken host reaction to Salmonella infection. Similarly, in the IDD group the time (t-e) category further indicated additional significant results especially for the phosphoprotein biological function.

Finally, a cluster analysis was performed for the lists of biological functional annotations. The DE-group (e) and (et) categories showed over 600 clusters. Due to the fact that the list of the (t) category in the DE group was longer than 3000 entries, clustering was technically not possible for the DAVID software. Since the lists of functional annotations of the (et) and the (t) categories of the DE group were very similar the (t) category clusters were deduced from the (e) and (et) categories clusters (see below).

Table 3 shows the results for clusters with enrichment scores larger than 2. An enrichment score indicate whether the number of genes in a cluster is equal to the expected number of genes (due to the number of genes of that physiological group in the genome and on the microarray) or higher or lower than expected. A high enrichment score thus indicates that the physiological trait of the cluster may be significant to the trait. The clusters were ordered by enrichment scores. The range of enrichment scores was from over 10 to almost zero. Enrichment scores less than 2 were omitted, leaving between 35 and 40 clusters in the DE-group (e) and (et) categories, respectively. For completeness, the Additional file 3 includes the biological functions per cluster in detail, i.e. larger than 1. The content of each cluster is a group of biological functions taken together from various databases centered on a specific theme. For example (see Table 3), cluster 1 groups biological functions together related to the lumen of cell organelles, especially relating to the nucleus. Cluster 2 groups nucleotide binding and phosphorylation functions (especially serine and threonine phosphorylation), while cluster 3 groups mitochondrial membrane functionalities. The Table contains both cell / tissue morphological clusters and (macro) molecular biogenesis functional clusters. The functions of several protein domains were also relevantly clustered.

Taken together functional clustering analysis showed that these functional annotations can be grouped together in higher order biological morphological structures and biological processes. The clusters can be divided in 21 clusters describing metabolic processes – of which three were related to energy metabolism, eight clusters describing (cell) morphological features, three clusters specifically pointing to protein domains involved, one cluster related to apoptosis and one cluster is a collection of processes, making it difficult to recognize a central theme. Apart from apoptosis, these clusters describe normal cellular physiological processes taking also place in non-infected animals, e.g. during growth and development of the tissues and organs. Nevertheless, these processes also participate in the host reaction to infection with Salmonella.

Apart from small differences in the order of clusters the (e) and (et) categories of the DE group differ only in a few clusters from each other. A specific tyrosine phosphorylation was found in the (et) category but not in the (e) category of the DE group while the (e) category showed a protein domain WD cluster and a cell movement cluster, both not found in the (et) category. Finally, it should be noted that in none of the other categories (DE and IDD groups) a significant cluster with enrichment score of at least 2 could be found.

## Discussion

The objective of this study was to determine the general chicken host response to Salmonella infection independent of age of the chicken host at time of bacterial challenge and independent of host response time post-infection, investigating various tissues and using chicken lines differing in susceptibility for Salmonella. The results highlight several biological mechanisms related to energy metabolism, apoptosis, specific protein domains indicating groups of involved proteins, and several cellular morphological structures where the affected processes are taking place. Overall, the reported meta-analysis approach showed successful integration of heterogeneous data sets of limited size by increasing statistical power. Using the results of this study for future biomarker analysis may provide in early diagnosis and warning of potentially hazardous food.

### Meta-analysis using data from different sources and different technologies

A meta-analysis is performed using the original raw data from a number of individual experiments. Since the experiments may have different objectives and use different technologies, the experiments or data may not be directly comparable. In our study we compared data from four studies using: (1) genetically different chicken lines differing in Salmonella susceptibility), (2) different Salmonella serovars, (3) different sampling time points, (4) different sampled tissues, (5) different microarray types, and (6) different ages of bacterial challenge. Intuitively, it would be expected that these differences would affect the meta-analysis: (1) Genetically different lines of chicken, differing in Salmonella susceptibility, were expected to differ in reaction mechanism and/or reaction severity. (2) A pathogen specific host reaction was expected to different Salmonella serovars. (3) Sampling at different times post infection was suggested to show different temporal expression patterns related to the stage of infection. (4) Expression patterns are also expected to differ between different tissues or cell types. (5) Finally, different microarrays contained different sets of genes, so results from one study were expected to be missing from another study and vice versa. (6) The age of challenge of the birds would be expected to produce very different responses due to the poorly developed immune system of day old chicks compared to 3 week old birds. Despite of all these differences our meta-analysis indicated that the chicken lines react to Salmonella infection through comparable mechanisms irrespective of Salmonella serovar and tissue type, and therefore it may be concluded that we identified common mechanisms of the host response to the bacterial challenge. However, due to the different experimental ages of the animals used in the diverse studies, this conclusion may be hampered by the developmental differences of tissues and organs in the animals. It can be expected that at least part of the mechanisms found may relate to this. This could have been investigated only if control samples of all experimental ages in the individual datasets would have been available. But often these control samples are only available for the last experimental sampling age. Further experiments are needed to elucidate this point.

Although it is not certain, it can be expected that the results would have been more comprehensive if all experiments were performed under standard procedures. Similarly, the functional annotation analysis to elucidate potential biological mechanisms of the functional reaction of chicken to Salmonella infection would have been more robust.

### What does the functional annotation analysis teach us about the chicken host reaction to the infection with a Salmonella bacterium?

The first indication that the chicken host reaction to Salmonella infection was similar between the diverse experiments was obtained from the similarities in the gene lists for the differently conducted experiment (e) and time (t) categories, i.e. the (et) category. One unexpected finding was that the expression profiles related to several different cell types. Intestinal tissue is composed of many different cell types that could be indicated by a mixture of expression profiles. Furthermore, localized infection will change tissue expression profiles, which will be exacerbated by the influx of immune cells, which will further change the overall expression profile. However, the results indicated similarities to the expression profiles of several cell types including many unrelated tissues like liver and brain. The epithelium cell type of the intestine was the highest ranking tissue in all three analysis groups. Also platelet and muscle tissue, and perhaps colon expression profiles were recognizable – these cell types are also included in the intestinal tissue. Other cell types may also be on the list for several reasons. One reason may be that a cell type has a high turnover rate like epithelial cells in intestine tissue. In these cell types the general mechanism for cell division will be activated and therefore all these cell types appear on the list. Especially in developing young-age animals this may be expected. Finally, cell types and tissues may have been included in the list because we used the human physiological information instead of chicken physiological information for the DAVID software to create the list, e.g. lymph tissue may be inserted for that reason (although the chicken intestine contains a limited number of Peyer’s patches as lymphoid tissues [38]).

The functional annotation is the result of the analysis of the DAVID software using the same gene lists to analyze several different databases containing biological function information. Due to the similarities within the gene lists the DE group (e), (t), and (et) categories showed similar functional annotations. Furthermore, the top category functional annotation “phosphoprotein” was also found in the differently expressed genes unique for both the experiments and the time (t) array after infection, and in the time-related genes found specifically in the meta-analysis (IDD-group). These results suggest two mechanisms: (1) the analysis is robust and indicated the same biological functionalities for all experiments despite the experimental differences, and (2) the meta-analysis adds new genes and data to the already existing data, but does indicate new biological mechanisms for the reaction of chicken hosts to the Salmonella infection. On the other hand, protein phosphorylation is an important regulatory mechanism for protein function in normal tissue and changed phosphoprotein content of the cell may have important physiological consequences for cellular metabolism (see below).

The clustering of biological functional annotations showed only in the lower part of the list differences between the DE (e) and DE (t) category. While these differences themselves were statistically significant, the place on the list may suggest that the differences in the reaction of chicken to Salmonella are small. Alternatively, these differences point towards differences in the expression profiles related to time point after infection. However, due to the structure of the dataset these differences may also relate to deviations in the general chicken reaction mechanism caused by different chicken breed/lines, different tissues or different Salmonella serovars (jejunum vs. caecum; *S.* Enteritidis vs. *S.* Typhimurium).

While most clusters of differently expressed genes were similar in both the experiment (e) and the time (t) categories, some interesting differences were obtained. It should be noted that clusters found in one category but not in the other may be the result of real missing clusters or clusters failing to reach the enrichment score limit in one of the two categories. Three clusters were found in the differently expressed genes group experiment (e) category, but not in the time (t) category suggesting that these genes were not, or less regulated in time after infection and may be constitutively active during the chicken host reaction to Salmonella infection: (E1) Tyrosine phosphorylation, (E2) Protein domain WD (repeat), and (E3) cell movement. Two clusters were found in the differently expressed genes group time (t) category but not in the experiment (e) category, suggesting that these genes were especially regulated at different moments in time after infection of the chicken: (T1) ubiquitin / proteasome mediated proteolysis, and (T2) Intracellular vesicles.

#### Tyrosine phosphorylation (E1)

Phosphorylation activates or deactivates many proteins in cellular processes and protein phosphorylation in particular plays a significant role in a wide range of cellular processes [39-41]. Tyrosine phosphorylation is considered to be one of the key steps in signal transduction and regulation of enzymatic activity (for a review see [42]. The consequences of the difference between the (e)- and (t) categories (for tyrosine phosphorylation) may be important.

Both signal transduction and enzymatic activity may regulate a variety of important processes in the cell, including immune processes, cellular metabolism, and cell morphology, which may be related to the chicken host reaction to infection with Salmonella, e.g. via changes in the actin cytoskeleton [43,44].

#### Protein domain WD (E2)

The WD40 repeat (also known as the WD or beta-transducin repeat) is a short structural motif of approximately 40 amino acids, often terminating in a tryptophan-aspartic acid (W-D) dipeptide [45]. Several of these repeats are combined to form a type of protein domain called the WD domain. WD-containing proteins have 4 to 16 repeating units, all of which are thought to form a circularized beta-propeller structure [46,47]. WD-repeat proteins are a large protein family found in all eukaryotes and are implicated in a variety of functions ranging from signal transduction and transcription regulation to cell cycle control and apoptosis, which may be directly related to the chicken immune reaction to the Salmonella infection. All these specific functions were also found in other clusters. Thus, the difference between the (e) and (t) categories may induce modulations of the intensities of the processes described in several of the other clusters, thereby representing another mechanism for these proteins to modulate the chicken host response to Salmonella infection. Furthermore, the underlying common function of all WD-repeat proteins is coordinating multi-protein complex assemblies, where the repeating units serve as a rigid scaffold for protein interactions. The specificity of the proteins is determined by the sequences outside the repeats themselves. Several of the clusters relate to macromolecules which may be differently regulated between the (e) and (t) categories. A specific macromolecule includes the E3 ubiquitin ligase suggesting that also proteolysis is regulated [46,47].

#### Cell movement (E3)

Cell movement could relate to the influx of immune cells to the site of infection/ tissue. Also in non-infected tissues immune cells move through the tissue, but this process will be enhanced during infection. It may be suggested that regulation of this process may be one of the fundamental mechanisms of the cellular immune response of the chicken host.

#### Ubiquitin / proteasome mediated proteolysis (T1)

The destination of Ubiquitin tagged proteins is the proteasome for proteolysis. The ubiquination system functions in a wide variety of cellular processes, including the immune response and inflammation, antigen processing, apoptosis and cell cycle. Furthermore, the development and degeneration of several tissues is affected – probably via biogenesis of organelles such as ribosomes and modulation of cell surface receptors, ion channels, and the secretory pathway (for a review see [48]. The ubiquination system is responsive to stress and extracellular modulators such as Salmonella infection [49]. It is clear that the wide variety of cellular metabolic functions regulated by the ubiquitin / proteasome system may affect the chicken host response to Salmonella. Its regulation of expression especially at different time points after infection can modulate the response of the chicken host to Salmonella infection through a variety of mechanisms described in the other clusters.

#### Intracellular vesicles (T2)

Intracellular vesicles transport material – e.g. (macro) molecules - through the cell – either importing or exporting material, or transporting material to different cellular locations. They deliver molecules both for excretion or to lysosomes for degradation, and may import food components for energy and cellular components synthesis processes. Lotz et al. [50] described that the HSP90 protein is important for the regulation of intracellular vesicle transport. The HSP90 protein is a molecular chaperone regulating the folding and thereby the activity of macromolecules [51]. These functions can be found in several of the other clusters. Thus, the differential expression especially at different time points after infection can modulate the response of the chicken host to Salmonella infection during the cause of the infection through a variety of mechanisms described in the other clusters.

## Conclusions

These results shed light on the important biological mechanisms that are active in the chicken gut cells during Salmonella infection – although part of the processes may relate to growth and development of the tissues and organs as discussed above. From our data we conclude that similar host mechanisms apply to *S.* Enteritidis and S. Typhimurium infection, and that similar biological mechanisms appear underlying the processes regulated during different times after infection.

## Abbreviations

DE = The Number of Differentially Expressed genes; IDD = (Integration Driven Discoveries): the number of genes that were determined differentially expressed in the meta-analysis that were not identified in any of the individual studies alone (i.e. new differentially expressed genes); e = Experiment - i.e. 4 studies; t = Time after infection – 14 different time point post-infection; et = Overlap between the (e) and (t) groups; e-t = Genes unique in (e); t-e = Genes unique in (t).

## Competing interests

The authors declare that they have no competing interests

## Authors’ contributions

MtP: Coordinated the meta-analysis, participated in the design of the meta-analysis, participated in the analysis of the results of the meta-analysis, and drafted the manuscript. IH: Participated in the design of the meta-analysis, performed the pre-processing of the data, participated in the meta-analysis, and helped in the analysis of the results of the meta-analysis. DS: Carried out the molecular genetic studies, analyzed the molecular results, participated in the design of the meta-analysis, participated in the meta-analysis, and helped in the analysis of the results of the meta-analysis. MS: Participated in the coordination the overall work in the project, participated in the coordination of the meta-analysis, participated in the design and analysis of the meta-analysis, and participated in drafting the manuscript. MF: Carried out the molecular genetic studies, analyzed the molecular results, and participated in drafting the manuscript. RZ: Carried out the animal studies, molecular genetic studies and immunologic reactions. M-LE: Carried out the animal studies, molecular genetic studies and immunologic reactions. AR: Coordinated the overall work in the project, participated in the coordination of the meta-analysis, participated in the design and analysis of the meta-analysis, and participated in drafting the manuscript. All authors read and approved the final manuscript.

## Supplementary Material

Additional file 1 The lists of genes with regulated expression in more than one or all experiments of the meta-analysis DE-group. Click here for file

Additional file 2 Functional biological mechanisms of the differently expressed genes. Click here for file

Additional file 3 Details of the biological functions per cluster. Click here for file
